# *MAOA* rs1137070 and heroin addiction interactively alter gray matter volume of the salience network

**DOI:** 10.1038/srep45321

**Published:** 2017-03-27

**Authors:** Yan Sun, Linwen Liu, Jiajia Feng, Weihua Yue, Lin Lu, Yong Fan, Jie Shi

**Affiliations:** 1 National Institute on Drug Dependence, Peking University, Beijing 100191, China; 2 National Laboratory of Pattern Recognition, Institute of Automation, Chinese Academy of Sciences, Beijing 100190, China; 3 Institute of Mental Health/Peking University Sixth Hospital and Key Laboratory of Mental Health, Peking University, Beijing 100191, China; 4 Department of Radiology, Perelman School of Medicine, University of Pennsylvania, Philadelphia, PA 19104, USA; 5 Beijing Key Laboratory on Drug Dependence Research, Beijing 100191, China; 6 The State Key Laboratory of Natural and Biomimetic Drugs, Beijing 100191, China

## Abstract

The rs1137070 polymorphism of monoamine oxidase A (*MAOA*) is associated with alcoholism and smoking behavior. However, the association between rs1137070 and heroin addiction remains unclear. In this study, we examined the allelic distribution of rs1137070 in 1,035 heroin abusers and 2,553 healthy controls and investigated the interactive effects of rs1137070 and heroin addiction on gray matter volume (GMV) based on 78 heroin abusers and 79 healthy controls. The C allele frequency of rs1137070 was significantly higher in heroin abusers. Heroin addiction and the rs1137070 variant interactively altered measures of GMV in the anterior cingulate cortex, orbital frontal cortex, temporal pole, and insula, which were correlated with cognitive function. Heroin abusers with the C allele had lower measures of GMV in these regions than the healthy controls with the same allele, whereas those with the T allele displayed a different trend. The altered brain regions were connected with white matter tracts, yielding a structural network that partially overlapped with the salience network. These findings suggest that the low activity-related C allele of *MAOA* rs1137070 is associated with an increase in the sensitivity to heroin addiction and the damaging effects of heroin abuse on cognition and the salience network.

Drug addiction is characterized by uncontrollable drug intake and drug-seeking behavior. It is a chronic psychiatric disorder with serious consequences, such as loss of productivity, social marginalization, and criminality^1^. Impulsivity is both an important facilitator of drug abuse and further impaired by drug addiction itself, which subsequently results in more impulsive drug intake behavior^2^. Drug abuse can result in persistent brain changes in molecular, morphology, and neural circuits that are critical for emotional regulation and cognitive control^3^.

Genetic factors contribute 40–60% to the development of addiction and operate at all stages of addiction^4^. The monoamine oxidase A (*MAOA*) gene is an X-linked gene that has been associated with various psychiatric disorders^5^, including addiction^6^,^7^, Monoamine oxidase A metabolizes and regulates biogenic amines, such as dopamine, norepinephrine, serotonin, and histamine at monoaminergic synapses in the brain^8^. The activity levels of *MOAO* vary widely among individuals^9^. The activity of *MAOA* is crucial for early brain development and has long-term effects on human behavior that may play an important role in the pathogenesis of addiction^10^. Animal studies have shown that *MAOA* knockout mice exhibit impairments in nicotine preference but normal responses to novel stimuli^11^. Therefore, *MAOA* is an important candidate gene that is responsible for interindividual differences in the susceptibility to substance addiction.

The rs1137070 polymorphism of *MAOA* is a restriction endonuclease site that is located at position 1460 in the *MAOA* gene. The C allele of rs1137070 lacks a restriction endonuclease site and is associated with lower *MAOA* activity^12^. This genetic variation is associated with various psychiatric disorders^5^, including alcohol dependence^6^. Smoking behavior is also affected by rs1137070, suggesting a possible contribution of this variant to nicotine dependence^7^. However, the association between rs1137070 and heroin addiction remains unclear.

We examined the association between *MAOA* rs1137070 and heroin addiction. We first measured the genetic distribution of the rs1137070 polymorphism based on a large sample of heroin abusers and normal controls and then evaluated its effects on gray matter volume (GMV) and neurocognitive performance in a subgroup of subjects with imaging data.

## Results

### Characteristics of subjects, neurocognitive performance, and gray matter volume

The demographic and addictive characteristics of the genetic sample are shown in Table S1. For males, females, and the entire cohort, statistically significant differences in age were found between heroin abusers and healthy controls. In the subset of subjects with imaging data, cigarette use was higher in heroin abusers than in controls. Heroin abusers had significantly higher impulsiveness scores on the Barratt Impulsiveness Scale (BIS-11) and worse performance on the Montreal Cognitive Assessment (MoCA) and Iowa Gambling Task (IGT; Table S2). Compared with healthy controls, widespread gray matter (GM) atrophy was found in heroin abusers (Fig. S1).

### Distribution of rs1137070 allele frequencies

The genotype frequencies did not deviate from Hardy-Weinberg equilibrium (control: *p* = 0.102; heroin: *p* = 0.188). Significant differences in the allele frequencies of *MAOA* rs1137070 were found between heroin abusers and healthy controls in males, females, and the entire cohort. The C allele frequency of rs1137070 was significantly higher in heroin abusers than in healthy controls (male: *p* = 0.015; female: *p* = 0.015; total: *p* < 0.001; Table 1).

### Effects of rs1137070 on gray matter volume

**Figure 1:**
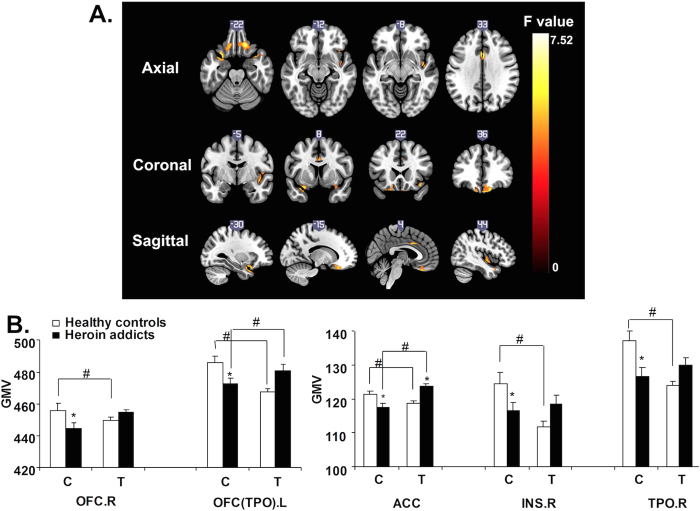
Interactive effects of *MAOA* rs1137070 and heroin addiction on gray matter volume (GMV). (**A**) Brain areas with statistical significance were the left orbital frontal cortex and temporal pole (OFC [TPO].L), right orbital frontal cortex (OFC.R), bilateral anterior cingulate cortex (ACC), right insula (INS.R), and right temporal pole (TPO.R). (**B**) Compared with T allele carriers, C allele carriers presented an increase in GMV compared with healthy controls, but a significant decrease in GMV was observed in heroin abusers. C allele carriers with heroin addiction exhibited a significant decrease in GMV in these areas compared with healthy C allele carriers. In T carriers, heroin abusers exhibited no changes in GMV, with the exception of the ACC, which exhibited a marked increase in GMV compared with healthy controls. No significant difference was found between C allele heroin abusers and T allele controls or between T allele heroin abusers and C allele controls. *p < 0.05, compared with healthy controls in each genotype; ^#^*p* < 0.05, compared with the other genotype of heroin abusers and healthy controls. Error bars indicate standard errors.

**Figure 2:**
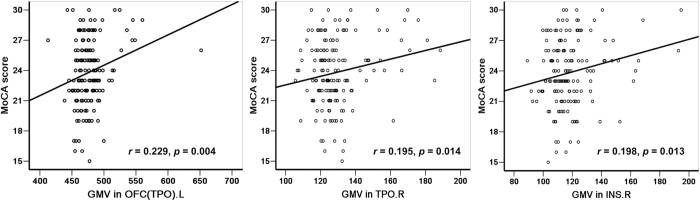
Positive correlations between Montreal Cognitive Assessment (MoCA)-assessed cognitive ability and gray matter volume (GMV) in brain areas affected by the interaction between addiction and rs1137070. The affected areas included the left orbital frontal cortex and temporal pole (OFC [TPO].L), right temporal pole (TPO.R), and right insula (INS.R).

The rs1137070 variant interacted with heroin addiction to affect GMV in the bilateral orbital frontal cortex (OFC), bilateral temporal pole (TPO), bilateral anterior cingulate cortex (ACC), and right insula (INS.R; Fig. 1, Table 2). The C allele and T allele differentially affected these regions in heroin abusers and healthy controls. Heroin abusers with the C allele had lower measures of GMV than healthy controls with the same allele, whereas those with the T allele displayed a different trend. Most of the regional GMV differences were statistically significant, as shown in Fig. 1B. Moreover, the measures of GMV in the OFC (TPO).L, and INS.R were positively correlated with cognitive scores on the MoCA (Fig. 2).

**Figure 3:**
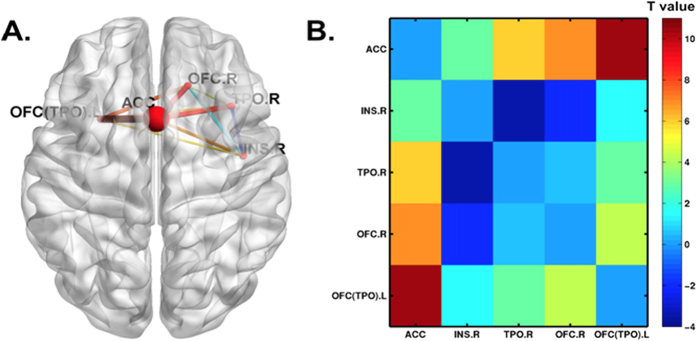
Fractional anisotropy (FA)-weighted white matter network connectivity among the areas that were affected by the addiction × rs1137070 interaction. (**A**) The ACC-OFC.R, ACC-TPO.R, ACC-INS.R, ACC-OFC (TPO).L, OFC (TPO).L-TPO.R, and OFC (TPO).L-OFC.R connections significantly increased in heroin abusers (shown in red), and the INS.R-TPO.R connection significantly decreased in heroin abusers (shown in blue). The ACC had the most significant connections with the other regions. (**B**) T values of each connection in this network. OFC (TPO).L, left orbital frontal cortex and temporal pole; OFC.R, right orbital frontal cortex; TPO.R, right temporal pole; INS.R, right insula; ACC, bilateral anterior cingulate cortex. The *p* value for each connection is presented in Table S3.

A structural connectivity analysis further revealed that the heroin abusers and healthy controls had different structural connections among these brain regions, measured by mean fractional anisotropy (FA) of white matter fiber tracts. Heroin abusers had stronger ACC-OFC.R, ACC-TPO.R, ACC-INS.R, ACC-OFC (TPO).L, OFC (TPO).L-TPO.R, and OFC (TPO).L-OFC.R connections and a weaker INS.R-TPO.R connection compared with healthy controls. Interestingly, the ACC was a hub node that was significantly connected with all of the other regions (Table S3, Fig. S2, Fig. 3).

## Discussion

The present study investigated the association between the *MAOA* rs1137070 polymorphism and heroin addiction and related brain morphological and neurocognitive abnormalities. The results showed that the *MAOA* rs1137070 had an allelic association with heroin addiction. Furthermore, rs1137070 and heroin addiction interactively contributed to GMV alterations in the OFC, TPO, ACC, and INS.R. These affected brain areas are related to cognition and partially overlap with the salience network^13^,^14^. The C allele was associated with increased susceptibility to heroin addiction and GM loss in heroin abusers.

The low-activity C allele of *MAOA* rs1137070 was associated an increased susceptibility to heroin addiction. The rs1137070 polymorphism is a synonymous variant, and allelic differences at this position do not alter the amino acid sequence but rather affect the presence or absence of restriction sites and consequent levels of *MAOA* activity^12^. Differences in *MAOA* activity levels were correlated with the number of active enzyme molecules^15^, which can result in the same effects as the regulation of expression. The effects of this low-activity allele of *MAOA* rs1137070 were similar to the low-expression variant of the upstream variable number tandem repeat region in the *MAOA* promoter, which was shown to increase the risk for alcohol addiction^6^, GM atrophy in cocaine abusers^16^, and antisocial-violent behavior in heroin abusers^17^.

*MAOA* rs1137070 had neural system-level effects on GM. In the present study, a subset of male subjects were scanned to obtain imaging data because the allelic effect can be assessed more clearly in male subjects who carry a single X chromosome. We found that the decrease in *MAOA* activity interacted with addictive drug use to decrease GMV, which is consistent with a previous study^16^. However, the mechanisms by which this occurs remain unknown.

*MAOA* is predominantly localized in catecholaminergic neurons in the primate brain^18^ and preferentially oxidizes serotonin, norepinephrine, and dopamine^8^. The inhibition of *MAOA* activity increases the actions of dopamine and other monoamine neurotransmitters that are responsible for drug reinforcement and motivation^8^, thus exacerbating the pathological changes in the brain that are induced by addictive drugs^3^. The modulatory effect of a specific *MAOA* genotype on the brain begins in early brain development and may be augmented by drug addiction. We postulated that epigenetic modifications might contribute to the impact of *MAOA* on GM. Notably, the brain areas that were altered, including the OFC, TPO, INS, and ACC, are predominantly involved in cognitive function^19^. Drug abusers who carry the C allele of rs1137070 may show more serious deficits in cognition that can deteriorate into compulsive drug-taking behavior. *MAOA* rs1137070 was associated with both the risk of onset of heroin addiction and also its progression.

The present study also found that the brain regions that were interactively affected by rs1137070 and heroin use were connected by white matter tracts, thus yielding a structural network with the ACC as the hub node. The function of the anterior insula and ACC is to segregate the most relevant stimuli among internal and external stimuli to guide behavior, and this function is altered in addiction^13^,^14^. The ACC also plays a critical role in cognitive control and impulsivity in abusers^20^. The connections among these altered brain regions may represent a structural basis for disruptions in the cognitive function network. This purported brain network may represent a biomarker of addiction and provide new therapeutic targets for MAO-related drugs to treat addiction^21^.

The present study was limited by its cross-sectional design; therefore, we could not differentiate the effects of genetic factors during different stages of addiction. The further follow-up study about the rs1137070 effects on drug relapse is desirable. We also did not collect functional magnetic resonance imaging (MRI) data and thus were unable to investigate functional brain networks, although we identified abnormal brain regions that partially overlapped with the salience network.

In conclusion, we found that the *MAOA* rs1137070 was significantly associated with the susceptibility to heroin addiction and changes in GM. The low activity-related C allele of rs1137070 was associated with an increased risk of heroin addiction and the deleterious effects of heroin abuse on GM. The brain areas that were found to be altered are involved in neurocognitive performance, and *MAOA* rs1137070 may play an important role in impaired cognition in heroin abusers. The function of the ACC-centered network may be critical for mediating the genetic effect of *MAOA* rs1137070 on heroin addiction.

## Methods

### Subjects

We recruited 1,035 heroin abusers (725 males, 307 females) from multiple drug addiction treatment centers in China’s Guangdong and Hubei provinces and 2,553 healthy controls (1,113 males, 1,440 female) from local communities. Only Han Chinese whose parents were native to southern China were included in the study. Inclusion criteria for the heroin group were the following: met the *Diagnostic and Statistical Manual of Mental Disorders*, 4th edition, criteria for opiate dependence and had no history of poly-substance abuse (continuous use of other opioid drugs for no more than 1 month, use of other kinds of addictive drugs no more than three times), with the exception of nicotine dependence. The subjects had been abstinent from heroin for less than 2 years at the time of the study. Healthy subjects were excluded if they had any history of substance abuse or dependence other than nicotine. All of the subjects had no past or current major medical conditions and no major psychiatric disorders other than substance addiction (and no family history of these). Alcohol abusers were also excluded based on the Michigan Alcoholism Screening Test (≤4 points).

Magnetic resonance imaging scans and neurocognitive performance were examined in a subset of the initial sample that consisted of 78 male heroin abusers and 79 male healthy controls (C allele: 39 controls and 36 cases; T allele: 40 controls and 42 cases) who had an ability to understand Mandarin and a level of education that was higher than primary school. Additional exclusion criteria that were applied for subjects who participated in the imaging study included left-handedness and contraindications for the MRI environment (e.g., claustrophobia, dentures, head trauma, and metal implants).

This study was approved by the Peking University Institutional Review Board, and the experiment was performed in accordance with relevant guidelines and regulations. The subjects signed a written informed consent form and were paid for their participation.

### Genotyping

Genomic DNA was extracted from approximately 200 μl peripheral blood samples using the QIAamp DNA Mini Kit (Qiagen, Hilden, Germany). The single-nucleotide polymorphism was genotyped using the Sequenom Mass Array system (SequenomiPLEX assay). Locus-specific polymerase chain reaction was performed on the DNA samples, and the detection primers were designed using MassARRAY Assay Design 3.0 software (Sequenom, San Diego, CA, USA). The resulting products were then desalted and transferred to a 384-element SpectroCHIP array, and matrix-assisted laser desorption/ionization-time of flight mass spectroscopy was used for allele detection. The mass spectrograms were analyzed using MassARRAY TYPER software (Sequenom).

### Magnetic resonance imaging acquisition and image processing

The MRI data were acquired using a GE Signa Twin speed MRI 1.5 T scanner at Zhongshan Traditional Chinese Medicine Hospital. The subjects laid supine with their heads snugly fixed by straps and foam pads to minimize head movements. T1-weighted, sagittal three-dimensional images were acquired with a spoiled gradient recalled echo sequence that covered the entire brain (slice thickness = 1 mm, repetition time = 7.816 ms, echo time = 2.984 ms, inversion time = 450 ms, flip angle = 13°, acquisition matrix = 256 × 256, field of view = 256 × 256 mm^2^, number of averages = 2). T2-weighted images were also acquired to exclude subjects with abnormal brain images, which were checked by two experienced neuroradiologists. Diffusion-weighted images were acquired using a single-shot twice-refocused echo planar imaging sequence (56 axial slices, 2.4 mm slice thickness without gap, repetition time = 14.4 s, echo time = 85 ms, 25 non-linear directions with b = 1000 s/mm^2^, three additional images without diffusion weighting [b = 0 s/mm^2^], acquisition matrix = 128 × 128, field of view = 256 × 256 mm^2^, number of averages = 2).

Structural MRI data were preprocessed using SPM8 (http://www.fil.ion.ucl.ac.uk/spm; accessed February 1, 2017). All T1 scans were segmented into GM, white matter, and cerebrospinal fluid using the New Segment toolbox^22^ and spatially normalized to a group template using the DARTEL image registration algorithm. The spatially normalized GM images were modulated by a Jacobian determinant of their corresponding deformation fields, corrected by their corresponding total intracranial volume, and smoothed with a 6-mm full-width at half-maximum isotropic Gaussian kernel.

Diffusion tensor imaging (DTI) data preprocessing was performed using the FMRIB Software Library 5.0.7 (Oxford Centre for Functional Magnetic Resonance Imaging of the Brain Software Library, www.fmrib.ox.ac.uk/fsl/; accessed February 1, 2017) using the following steps: eddy-currents and head motion correction, estimation of the diffusion tensors, and calculation of FA. Structural MRI scans were co-registered with their corresponding DTI b0 image so that ROIs that were defined in the structural MRI space could be transformed to DTI space.

A structural connectivity analysis was performed among given ROIs that were defined as brain regions whose GMVs were interactively altered by heroin addiction and *MAOA* rs1137070. Probabilistic tracking between each pair of ROIs, implemented by FMRIB’s diffusion toolbox with its default setting, was used to identify structural connectivity paths. The average FA value of voxels on the paths was used to measure structural connectivity between two ROIs.

### Neurocognitive assessments

Neurocognitive performance was assessed using the MoCA (higher scores indicated greater cognitive ability), BIS-11 (higher scores indicated greater impulsiveness), and IGT (higher scores indicated better decision-making ability). Additional details are provided in the Supplementary Material.

### Statistical analysis

Because *MAOA* is an X-linked gene, we separately analyzed the genetic association in each sex group, and only female subjects were used to perform the test of Hardy-Weinberg equilibrium. Deviation of the genotype counts from Hardy-Weinberg equilibrium was evaluated by the *χ*^*2*^ goodness-of-fit test. The level of significance was set at a two-tailed *p* < 0.05.

A voxelwise two-way analysis of variance (ANOVA) model was used to explore the interactive effects of genetic variants and addiction status on brain regions in which differences in GMV were found between heroin abusers and healthy controls, with age and cigarette use as regressors. AlphaSim (AFNI software, http://afni.nimh.nih.gov/pub/dist/doc/manual/AlphaSim.pdf; accessed February 1, 2017) with a corrected *p* < 0.05 and voxelwise threshold of *p* < 0.025 was used to correct for multiple comparisons, resulting in a cluster size >117 mm. Brain regions with statistically significant morphometric alterations that were derived from the ANOVA were used as ROIs in the structural connectivity analysis. Independent two-sample *t*-tests were then used to compare the structural connectivity measures between heroin abusers and healthy controls. *Post hoc* analyses were performed using SAS 9.0 software (SAS Institute, Cary, NC, USA). Correlation analyses were performed using SPSS 13.0 software.

## Additional Information

**How to cite this article**: Sun, Y. *et al*. *MAOA* rs1137070 and heroin addiction interactively alter gray matter volume of the salience network. *Sci. Rep.* **7**, 45321; doi: 10.1038/srep45321 (2017).

**Publisher's note:** Springer Nature remains neutral with regard to jurisdictional claims in published maps and institutional affiliations.

## Supplementary Material

Supplemental Material

## Figures and Tables

**Table 1:** Allele distribution of *MAOA* rs1137070 in heroin abusers and healthy controls.

	Group	Allele* (Frequency)		*χ*^*2*^	*p*	Odds ratio	95% confidence interval
		T	C				
Male	Heroin *n* = 725	407 (0.561)	318 (0.439)	5.88	0.015	1.264	1.046–1.529
	Control *n* = 1113	688 (0.618)	425 (0.382)				
Female	Heroin *n* = 307	355 (0.578)	259 (0.422)	5.9	0.015	1.245	1.043–1.486
	Control *n* = 1440	1816 (0.631)	1064 (0.369)				
Total	Heroin *n* = 1032	762 (0.569)	577 (0.431)	14.22	<0.001	1.274	1.122–1.443
	Control *n* = 2553	2504 (0.627)	1489 (0.373)				

^*^Male allele = n, Female allele = 2n, Total allele = Male allele + Female allele.

**Table 2:** Clusters interactively affected by heroin addiction and *MAOA* rs1137070.

Region	L/R	Abbreviation	Cluster size	*p*	Z	Peak (X, Y, Z)
Orbital frontal cortex/temporal pole	L	OFC (TPO).L	709	0.014	2.2	−28, 14, −22
Orbital frontal cortex	R	OFC.R	654	0.007	2.47	16, 32, −26
Temporal pole	R	TPO.R	216	0.023	2	38, 21, −15
Insula	R	INS.R	204	0.022	2.01	44, −6, −8
Anterior cingulate cortex	Bilateral	ACC	152	0.009	2.35	0, 14, 33
