# Supplementary material for: MAOA rs1137070 and heroin addiction interactively alter gray matter volume of the salience network
Source: Sci Rep. 2017 Mar 27;7:45321. doi: 10.1038/srep45321 (PMC5366902; doi:10.1038/srep45321)
Supplement: Supplemental Material [file srep45321-s1.doc]

Feb. 5, 2017, Revision for *Scientific Reports*

**Supplementary Material**

***MAOA* rs1137070 and heroin addiction interactively alter gray matter volume** **of the salience network**

**Yan Sun,^1^, * Linwen Liu,^2^, * Jiajia Feng,^1^ Weihua Yue,^4^ Lin Lu,^1, 4^**

**Yong Fan,^3, #^ Jie Shi,^1, 5, #^**

*^1^**National Institute on Drug Dependence, Peking University, Beijing 100191, China*

*^2^**National Laboratory of Pattern Recognition, Institute of Automation,* *Chinese Academy of Sciences, Beijing 100190, China*

*^3^**Department of Radiology, Perelman School of Medicine,* *University of Pennsylvania, Philadelphia, PA 19104, USA*

*^4^**Institute of Mental Health/Peking University Sixth Hospital and Key Laboratory of Mental Health,* *Peking University, Beijing 100191, China*

*^5^Beijing Key Laboratory on Drug Dependence Research, Beijing 100191, China*

*Equally contributed to this work

**Running title:** *MAOA* rs1137070 and heroin addiction

**^#^Correspondence:**

Prof. Jie Shi, National Institute on Drug Dependence, Peking University, No. 38, Xueyuan Road, Haidian District, Beijing, China. Email: shijie@bjmu.edu.cn

or

Prof. Yong Fan, Department of Radiology, Perelman School of Medicine, University of Pennsylvania, Philadelphia, PA 19104, USA. Email: yong.fan@ieee.org

**This file includes:**

Supplementary Methods

Supplementary Table S1-S3

Supplementary Figure S1-S2

**Supplementary Methods**

*Neurocognitive assessments*

*Montreal Cognitive Assessment (MoCA).* The MoCA[^1^](#_ENREF_1) is a 30-point test that takes approximately 10-15 min to administer and less than 1 min to score. Items on this test include short-term memory recall (involving two learning trials of five nouns and delayed recall after approximately 5 min), visuospatial ability (assessed by a clock-drawing task and three-dimensional cube copying task), and multiple aspects of executive function (mainly assessed by an alternation task adapted from the trail-making B task). Attention, concentration, working memory, language, and orientation to time and place are also involved in the scale. Higher MoCA scores indicate greater cognitive ability.

*Barratt Impulsiveness Scale (BIS-11).* The BIS[^2^](#_ENREF_2) is a 30-item self-report questionnaire that assesses impulsive personality traits in three dimensions: attention (inattention and cognitive instability), motor behavior (spontaneous actions), and non-planning (lack of forethought). Subjects were administered the Chinese version 11. Higher BIS-11 scores indicate greater impulsiveness.

*Iowa Gambling Task (IGT).* We used the original card version of the IGT to evaluate decision-making performance. In the task, participants are presented with four different decks of cards and asked to select one card at a time, with the goal of maximizing their "jackpot" over 100 card choices. Unbeknownst to the participant, two of the four decks (C and D) are “advantageous.” Gains from these decks are relatively modest, but losses are modest as well, so that choosing these decks consistently will result in an overall gain in the jackpot. The other two decks (A and B) are “disadvantageous.” Gains from these decks are high, but losses are even higher, so that choosing these decks consistently will result in an overall loss from the jackpot. The outcome measure for this task is the net score (total number of selections from the advantageous decks minus total number of selections from the disadvantageous decks; [C+D] - [A+B]). Healthy individuals gradually switch their preference toward the advantageous decks and away from the disadvantageous decks. Subjects with better decision-making ability score higher.

Table S1. Characteristics of the genetic sample.

| **Characteristics** | **Male** **genetic sample** | | |  | **Female genetic sample** | | |  | **Total genetic sample** | | |
| --- | --- | --- | --- | --- | --- | --- | --- | --- | --- | --- | --- |
|  | Case | Control | *p* |  | Case | Control | *p* |  | Case | Control | *p* |
|  | *n* = 725 | *n* = 1113 |  |  | *n* = 307 | *n* = 1440 |  |  | *n* = 1035 | *n* = 2553 |  |
| Age (years) | 35.47 ± 6.61 | 31.19 ± 9.87 | < 0.001 |  | 36.19 ± 6.85 | 31.55 ± 9.71 | < 0.001 |  | 35.69 ± 6.69 | 32.24 ± 9.22 | < 0.001 |
| Gender (male/female) | NA | NA | NA |  | NA | NA | NA |  | 725/307 | 1113/1440 | < 0.001 |
| Cigarettes per day | 24.37 ± 12.32 | ND | ND |  | 23.16 ± 17.72 | ND | ND |  | 24.00 ± 14.13 | ND | ND |
| Heroin dosage (g/day) | 0.63 ± 0.69 | NA | NA |  | 0.71 ± 0.60 | NA | NA |  | 0.66 ± 0.66 | NA | NA |
| Abstinence time (months) | 7.88 ± 5.98 | NA | NA |  | 10.82 ± 6.71 | NA | NA |  | 8.85 ± 6.38 | NA | NA |
| Addiction duration (years) | 12.11 ± 6.29 | NA | NA |  | 10.82 ± 6.33 | NA | NA |  | 11.72 ± 6.33 | NA | NA |

The data are expressed as mean ± standard deviation. NA, not applicable; ND, no data.

Table S2. Subject characteristics and neurocognitive performance.

| **Item** | **Heroin abusers** | **Healthy controls** | ***p*** |
| --- | --- | --- | --- |
|  | (*n* = 78) | (*n* = 79) |  |
| Age | 36.33 ± 3.94 | 37.52 ± 4.98 | 0.10 |
| Cigarettes per day | 26.00 ± 9.09 | 8.72 ± 9.81 | < 0.001 |
| Heroin dosage (g/day) | 0.52 ± 0.35 | NA | NA |
| Abstinence time (months) | 5.39 ± 3.44 | NA | NA |
| Addiction duration (years) | 15.10 ± 3.59 | NA | NA |
| MoCA | 21.92 ± 2.54 | 25.70 ± 2.88 | < 0.001 |
| IGT | -4.63 ± 21.07 | 4.02 ± 23.82 | 0.015 |
| BIS-11 (Attention) | 16.90 ± 2.72 | 15.91 ± 2.29 | 0.015 |
| BIS-11 (Motor) | 23.83 ± 4.47 | 21.08 ± 3.77 | < 0.001 |
| BIS-11 (Non-planning) | 27.40 ± 5.36 | 24.71 ± 5.02 | 0.001 |
| BIS-11 (Sum) | 68.13 ± 9.08 | 61.70 ± 8.75 | < 0.001 |

The data are expressed as mean ± standard deviation. MoCA, Montreal Cognitive Assessment; IGT, Iowa Gambling Task; BIS, Barratt Impulsiveness Scale.

Table S3. *P* value for each connection.

| **Addiction group** |  | **ACC** | **INS.R** | **TPO.R** | **OFC.R** | **OFC (TPO).L** |
| --- | --- | --- | --- | --- | --- | --- |
|  | **ACC** | 0 | < 0.00001 | < 0.00001 | < 0.00001 | < 0.00001 |
|  | **INS.R** | < 0.00001 | 0 | < 0.00001 | < 0.00001 | < 0.00001 |
|  | **TPO.R** | < 0.00001 | < 0.00001 | 0 | < 0.00001 | < 0.00001 |
|  | **OFC.R** | < 0.00001 | < 0.00001 | < 0.00001 | 0 | < 0.00001 |
|  | **OFC (TPO).L** | < 0.00001 | < 0.00001 | < 0.00001 | < 0.00001 | 0 |
| **Healthy group** |  | **ACC** | **INS.R** | **TPO.R** | **OFC.R** | **OFC (TPO).L** |
|  | **ACC** | 0 | < 0.00001 | < 0.00001 | < 0.00001 | < 0.00001 |
|  | **INS.R** | < 0.00001 | 0 | < 0.00001 | < 0.00001 | < 0.00001 |
|  | **TPO.R** | < 0.00001 | < 0.00001 | 0 | < 0.00001 | < 0.00001 |
|  | **OFC.R** | < 0.00001 | < 0.00001 | < 0.00001 | 0 | < 0.00001 |
|  | **OFC (TPO).L** | < 0.00001 | < 0.00001 | < 0.00001 | < 0.00001 | 0 |
| **Differences between two groups** |  | **ACC** | **INS.R** | **TPO.R** | **OFC.R** | **OFC (TPO).L** |
|  | **ACC** | 0 | 0.00403 | < 0.00001 | < 0.00001 | < 0.00001 |
|  | **INS.R** | 0.00403 | 0 | 0.00117 | 0.06040 | 0.15536 |
|  | **TPO.R** | < 0.00001 | 0.00117 | 0 | 0.52264 | 0.00345 |
|  | **OFC.R** | < 0.00001 | 0.06040 | 0.52264 | 0 | 0.00004 |
|  | **OFC (TPO).L** | < 0.00001 | 0.15536 | 0.00345 | 0.00004 | 0 |

OFC (TPO).L, left orbital frontal cortex and temporal pole; OFC.R, right orbital frontal cortex; TPO.L, left temporal pole; TPO.R, right temporal pole; INS.R: right insula; ACC, bilateral anterior cingulate cortex.


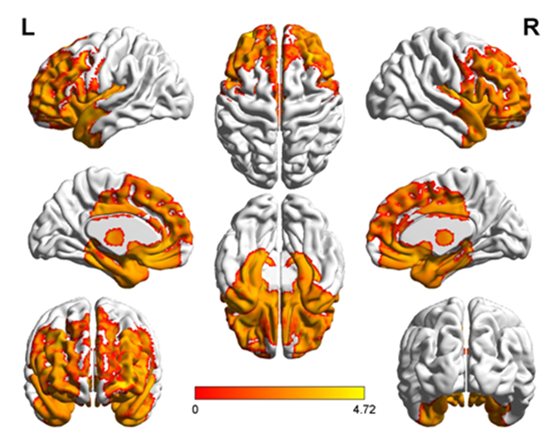


Figure S1. Regions with significant gray matter volume reductions in heroin abusers compared with healthy controls (FDR-corrected *p* < 0.05, cluster size > 100 voxels). The color-map indicates the scale for the *t*-statistic.


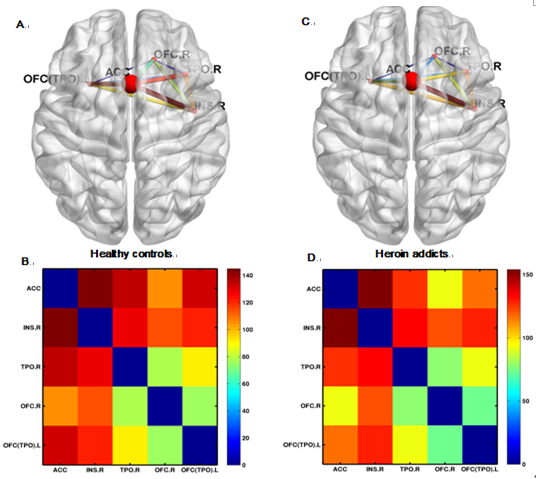


Figure S2. Fractional anisotropy-weighted white matter network of brain regions affected by the interaction between heroin addiction and rs1137070. (A) Connection with statistical significance in the healthy control group. (B) T value of each connection in the healthy control group. (C) Connection with statistical significance in the heroin addiction group. (D) T value of each connection in the heroin addiction group. The *P* value of each connection is shown in Table S3.

**Referenc**es

1. Luis, C.A., Keegan, A.P. & Mullan, M. Cross validation of the Montreal Cognitive Assessment in community dwelling older adults residing in the Southeastern US. *Int J Geriatr Psychiatry* **24**, 197-201 (2009).

2. Patton, J.H., Stanford, M.S. & Barratt, E.S. Factor structure of the Barratt Impulsiveness Scale. *J Clin Psychol* **51**, 768-74 (1995).

3. Bechara, A., Damasio, A.R., Damasio, H. & Anderson, S.W. Insensitivity to future consequences following damage to human prefrontal cortex. *Cognition* **50**, 7-15 (1994).

4. Bechara, A., Tranel, D. & Damasio, H. Characterization of the decision-making deficit of patients with ventromedial prefrontal cortex lesions. *Brain* **123**, 2189-202 (2000).
